# *Helicobacter pylori*-induced activation of β-catenin involves low density lipoprotein receptor-related protein 6 and Dishevelled

**DOI:** 10.1186/1476-4598-9-31

**Published:** 2010-02-05

**Authors:** Thorsten Gnad, Maria Feoktistova, Martin Leverkus, Uwe Lendeckel, Michael Naumann

**Affiliations:** 1 Institute of Experimental Internal Medicine, Otto von Guericke University, Leipzigerstraße 44, 39120 Magdeburg, Germany; 2 Laboratory of Experimental Dermatology, Department of Dermatology and Venerology, Otto von Guericke University, Leipzigerstraße 44, 39120 Magdeburg, Germany; 3 Institute of Medical Biochemistry and Molecular Biology, Ernst Moritz Arndt University, Klinikum Sauerbruchstraße, 17487 Greifswald, Germany

## Abstract

**Background:**

The human microbial pathogen *Helicobacter pylori *resides in the stomach of about fifty percent of the world's population and represents a risk factor for chronic gastritis, peptic ulcers and, in rare cases, gastric cancer. Alterations of the Wnt/β-catenin signaling pathway have been described in almost every human cancer disease, due to the regulation of target genes being involved in cell cycle control, differentiation, cell migration or stem cell control. Our study aimed to elucidate the role of proximal Wnt signaling components low density lipoprotein receptor-related protein 6 (LRP6) and Dishevelled (Dvl) in the activation of β-catenin early after infection of gastric epithelial cells with *H. pylori*.

**Results:**

Infection of gastric epithelial NCI-N87 cells with *H. pylori *induces rapid phosphorylation of the Wnt/β-catenin pathway co-receptor LRP6 independent of the cytotoxin-associated gene A (CagA) or vacuolating cytotoxin A (VacA). However, bacteria lacking a functional type 4 secretion system (T4SS) failed to induce LRP6 phosphorylation. Further, we identified proteins of the Dvl family, namely Dvl2 and Dvl3, which are involved in LRP6 phosphorylation. *H. pylori*-induced nuclear accumulation of β-catenin and its transcriptional activation, and expression of Wnt target genes are strongly reduced in stable knockdown cell lines deficient for LRP6, Dvl2 or Dvl3.

**Conclusion:**

We analysed the *H. pylori*-induced activation of Wnt-signaling factors and demonstrate for the first time that the canonical Wnt-signaling proteins LRP6 and Dvl2 and Dvl3 are involved in the regulation of β-catenin.

## Background

Persistent infection of the gastric mucosa by the human pathogen *H. pylori *is a leading cause for the development of gastroduodenal diseases like chronic gastritis, peptic ulcers, gastric adenocarcinoma or mucosa-associated lymphoid tissue (MALT) lymphoma [1]. Environmental factors and genetic diversity of bacterial strains and of the host all contribute to the multifaceted nature of the disease. However, the precise signaling pathways involved in the development of the described malignancies are not clearly defined. Activation of the Wnt/β-catenin signaling pathway has been described in about 30% of gastric cancer patients, often due to N-terminal mutations in β-catenin impairing its proper degradation [2,3]. Additionally, alterations in the E-cadherin/β-catenin cell adhesion complex frequently occur in gastric cancers [4,5] associated with increased nuclear localization of β-catenin. However, the exact role of *H. pylori *in the regulation of β-catenin remained unclear to date. β-catenin is a ubiquitously expressed protein with a dual role. On the one hand it is important in the establishment and maintenance of adherence junctions and, therefore, mediating cell-cell adhesion by connecting E-cadherin via α-catenin to the actin cytoskeleton [6]. On the other hand it acts as transcription factor upon forming heterodimers [7] together with lymphocyte enhancer factor/T cell factor (LEF/TCF). Among a number of target genes are c-myc, Axin2 or MMP-7 [8-10], which are involved in different cellular processes like cell cycle control or cell migration. Mutations that constitutively activate β-catenin signaling thus lead to the development of cancer very commonly [11]. In non-stimulated cells, the protein level of free β-catenin is kept low by a so-called destruction complex consisting mainly of the tumor suppressor adenomatous polyposis coli (APC) and Axin, which build a scaffold on which the serine/threonine kinases casein kinase 1α (CK1α) and glycogen synthase kinase 3β (GSK3β) function to phosphorylate β-catenin on N-terminal residues Ser-45 (CK1α) and Ser-33, Ser-37, and Thr-41 (GSK3β) [12]. This phosphorylation of β-catenin leads to polyubiquitinylation and subsequent degradation in the 26S proteasome [13]. Upon binding of Wnt ligands to their receptors of the Frizzled family of serpentine receptors and to their co-receptors LRP5/6, phosphorylation of β-catenin becomes impaired by a complex mechanism not fully understood yet. Activation of the phosphoprotein Dvl leads to a sequence of events in which the C-terminus of LRP6 becomes phosphorylated by GSK3β and CK1γ [14,15], and Axin together with Dvl translocates to the plasma membrane. As a consequence, the degradation complex is not longer functional and β-catenin translocates to and accumulates in the nucleus. Recently it was shown that infection of epithelial cells with *H. pylori *suppresses GSK3β activity via the PI3K/Akt pathway, inhibiting proper β-catenin degradation, inducing LEF/TCF transactivation, and upregulation of the β-catenin target gene cyclin D1 in a CagA- and T4SS-independent manner [16].

In the presented work we used human gastric epithelial cells NCI-N87 to study LRP6- and Dvl-dependent regulation of β-catenin after infection with *H. pylori*. We show that 1) infection leads to phosphorylation of LRP6 which is independent of CagA and VacA, but depends on a functional T4SS, 2) members of the Dvl family, namely Dvl2 and Dvl3, are involved in LRP6 phosphorylation 3) *H. pylori-*induced nuclear translocation of β-catenin, LEF/TCF transactivation and target gene expression are significantly reduced in cells with a stable knockdown of LRP6, Dvl2, or Dvl3.

## Results

### *H. pylori *induces LRP6 phosphorylation in a T4SS-dependent manner

**Figure 1:**
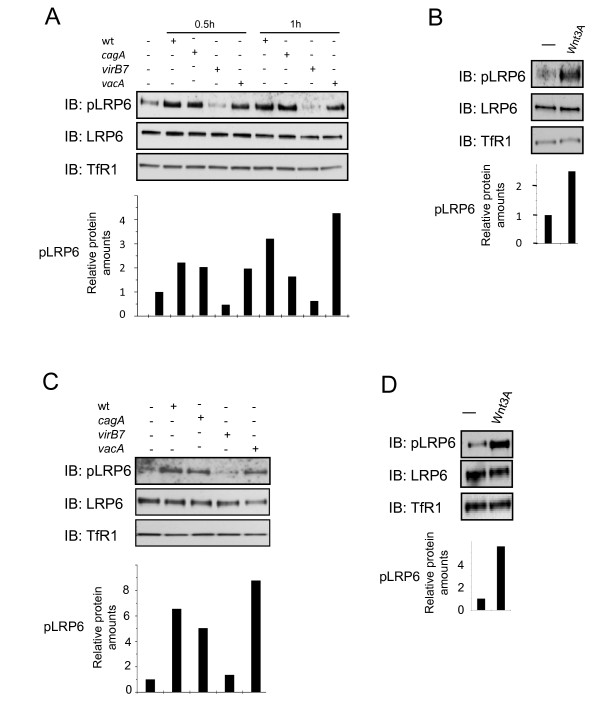
**Infection with *H. pylori *leads to LRP6 phosphorylation**. **A**, NCI-N87 cells were infected for 0.5 h or 1 h respectively with *H. pylori *P1 wildtype or mutant strains lacking CagA or VirB7. Additionally, cells were infected with a *H. pylori *P14 strain deficient for VacA. Membrane-enriched fractions were prepared as recommended by the manufacturer (S-PEK Kit, Calbiochem) and analysed by Western blot using the indicated antibodies. Diagrams indicate the relative amounts of pLRP6 after densitometric analysis. Densitometric values for pLRP6 were normalised to LRP6 and TfR1 in each sample and depicted relative to the values of non-infected cells. **B**, Western blot analysis of pLRP6 in NCI-N87 cells treated with recombinant Wnt3a (50 nM) for 0.5 h. **C**, AGS cells were infected for 0.5 h with *H. pylori *P1 wildtype or mutant strains lacking CagA or VirB7. Additionally, cells were infected with a *H. pylori *P14 strain deficient for VacA. Western blot analysis was performed as described in A. **D**, Western blot analysis of pLRP6 in AGS cells treated with recombinant Wnt3a (50 nM) for 0.5 h.

Phosphorylation of the co-receptor LRP6 is required for the Wnt/β-catenin signaling pathway, and LRP6 is indispensable for the stabilization of β-catenin. To study LRP6 phosphorylation in response to *H. pylori*, cells were infected with *H. pylori *P1 wild type or the isogenic mutant strains lacking the CagA protein or the virB7 protein (required for the integrity of the T4SS). In addition, a P14 strain lacking the VacA protein was used. Phosphorylation of LRP6 was induced within 30 minutes after infection in a CagA- and VacA-independent manner. However, a bacterial strain lacking a functional T4SS was not capable of inducing LRP6 phosphorylation (Figure 1A). To analyse putative cell type dependency, we studied whether *H. pylori *induces LRP6 phosphorylation in AGS cells. As in NCI-N87 cells, infection with *H. pylori *induced LRP6 phosphorylation (Figure 1C) independent of CagA or VacA, but required a functional T4SS. Treatment of NCI-N87 or AGS cells with recombinant Wnt3a induced LRP6 phosphorylation (Figure 1B, D). Wnt3a was chosen because it is a member of the canonical Wnt ligands of the Wnt-8 class. Members of this family are able eg, to transform epithelial mouse mammary gland cells which requires activated LRP6 and β-catenin [11].

### Phosphorylation of LRP6 requires Dvl proteins

**Figure 2:**
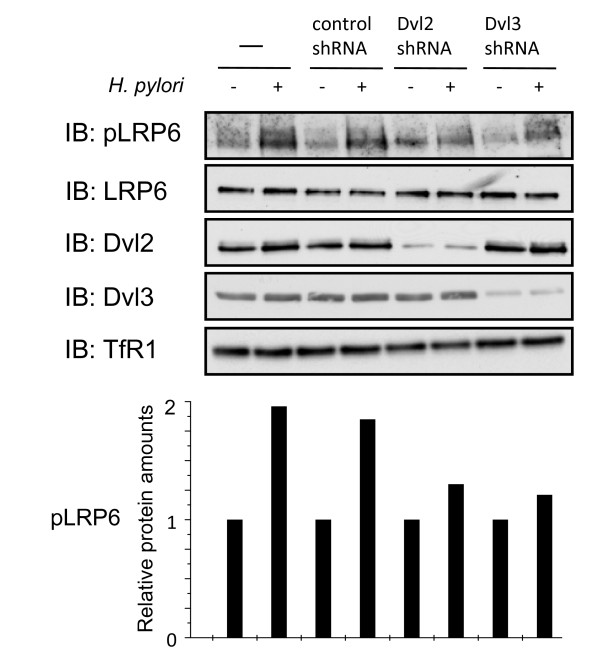
**LRP6 phosphorylation is reduced in Dvl2 and Dvl3 knockdown cell lines**. NCI-N87 cells, control shRNA cells, Dvl2 shRNA cells and Dvl3 shRNA cells were infected for 1 h with *H. pylori *P1 wildtype strain. Membrane-enriched fractions were prepared (S-PEK Kit, Calbiochem). Western blot analysis was performed with antibodies as indicated. Diagram indicates the relative amounts of pLRP6 after densitometric analysis. Densitometric values for pLRP6 were normalised to LRP6 and TfR1 in each sample and depicted relative to the values of non-infected cells.

Upon activation of the Wnt/β-catenin signaling pathway, receptors of the Frizzled family are thought to recruit the cytoplasmic phosphoprotein Dvl to the membrane. Dvl, in turn, recruits Axin and GSK3β to the membrane, the latter then phosphorylates LRP6 at serine 1490 [17]. Therefore, Dvl proteins are necessary for the stabilization of β-catenin. To study whether the phosphorylation of LRP6 after *H. pylori *infection depends on Dvl, we established stable NCI-N87 knockdown cell lines deficient for either Dvl2 or Dvl3. NCI-N87 cells, stably transfected with a scrambled shRNAmir, shDvl2mir or shDvl3mir were infected with *H. pylori *for 1 h. Subsequently membrane-enriched fractions were used for Western blot analysis using specific antibodies as indicated. Phosphorylation of LRP6 was reduced in cell lines deficient for Dvl2 and Dvl3, but not in non-treated parental NCI-N87 or mock transduced cells (Figure 2).

### *H. pylori *induces nuclear β-catenin accumulation, LEF/TCF activation, and target gene expression

**Figure 3:**
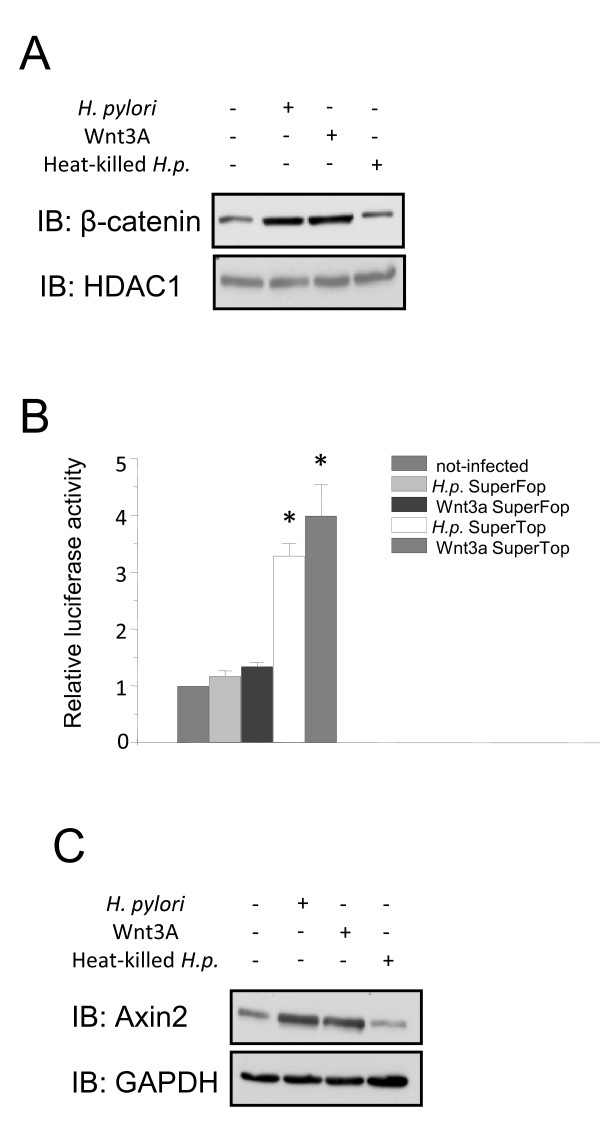
***H. pylori *induces nuclear β-catenin accumulation LEF/TCF activation and target gene expression**. **A**, NCI-N87 cells were infected for 1 h with *H. pylori *P1 wildtype strain or treated with recombinant Wnt3a or heat-killed bacteria (hk) for 1 h, respectively. Nuclear fractions were prepared (S-PEK Kit, Calbiochem). Western blot analysis was performed with antibodies as indicated. **B**, Cells were transiently transfected with either 8xSuperTOPflash or FOPflash and cotransfected with renilla luciferase plasmid. 24 h later, the cells were infected with *H. pylori *P1 wildtype strain or treated with recombinant Wnt3a, lysed and firefly/renilla luciferase activity was measured. Diagram shows the mean fold changes + SEM of 3 separate experiments performed in triplicate. * p < 0.01, relative to activity in non-infected cells. **C**, NCI-N87 cells were infected for 3 h with *H. pylori *P1 wildtype strain or treated with recombinant Wnt3a or heat-killed bacteria for 3 h. Cytosolic fractions were prepared (S-PEK Kit, Calbiochem). Western blot analysis was performed with the indicated antibodies.

Activation of the canonical Wnt signaling pathway leads to accumulation of β-catenin in the cytoplasm and subsequent translocation to the nucleus, resulting in target gene expression. As shown in figure 3A, infection with *H. pylori *led to an increase of β-catenin in the nuclear fraction within 1 h. Similarly, nuclear β-catenin was observed after treatment of NCI-N87 cells with recombinant Wnt3a. Importantly, heat-inactivated *H. pylori *did not induce nuclear translocation of β-catenin (Figure 3A) indicating that physical interaction of viable bacteria is required to induce nuclear β-catenin accumulation.

To investigate β-catenin-dependent gene transcription after infection with *H. pylori*, we performed a LEF/TCF reporter assay. NCI-N87 cells were transfected with luciferase reporter plasmid containing either eight copies of non-mutated (SuperTOPflash) or mutated (SuperFOPflash) LEF/TCF binding sites and then infected with *H. pylori*. After six hours of infection cells showed significant increased levels of luciferase activity in SuperTOPflash transfected cells compared to non-infected cells or SuperFOPflash transfected cells. Treatment of the cells with Wnt3a also increased reporter activity in SuperTOPflash transfected cells, but not in cells with the mutated LEF/TCF binding sites (Figure 3B). In order to investigate if transactivation activity induced *bona fide *target genes of Wnt, we analysed upregulation of the Wnt target gene Axin2. Cytosolic fractions were prepared and Western blot analysis was performed with an antibody specific for Axin2. Infection with *H. pylori *as well as treatment with recombinant Wnt3a, but not heat-killed *H. pylori *strongly induced protein expression of Axin2 (Figure 3C).

### *H. pylori*-induced nuclear accumulation of β-catenin, activation of LEF/TCF and target gene expression depend on LRP6, Dvl2 and Dvl3

**Figure 4:**
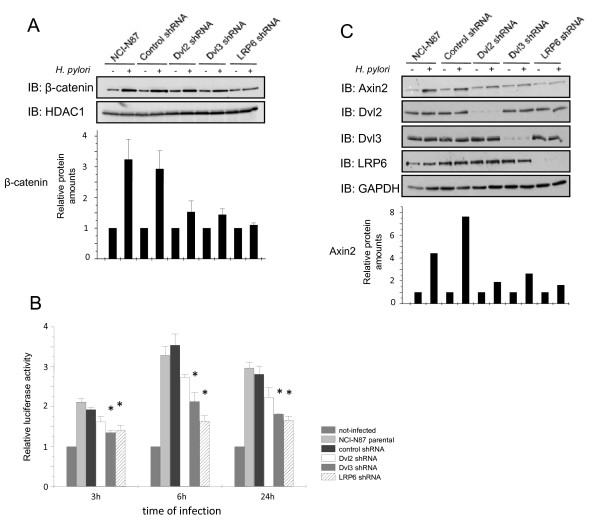
***H. pylori*-induced nuclear accumulation of β-catenin, activation of LEF/TCF and target gene expression depends on LRP6, Dvl2 and Dvl3**. **A **NCI-N87 cells, control shRNA, Dvl2 shRNA cells, Dvl3 shRNA cells, and LRP6 shRNA cells were infected for 1 h with *H. pylori *P1 wildtype strain. Nuclear fractions were prepared (S-PEK Kit, Calbiochem). Western blot analysis was performed with antibodies as indicated. Diagram shows the relative amounts of β-catenin after densitometric analysis from two independent experiments. Densitometric values for β-catenin were normalized to HDAC1 content in each sample and depicted relative to the values of the respective non-infected cells. **B**, Cells were transiently transfected with 8 × SuperTOPflash and cotransfected with renilla luciferase plasmid. 24 h later cells were infected with *H. pylori *P1 wildtype strain for the indicated periods of time, lysed and firefly/renilla luciferase activity was measured. Diagram represents the mean fold changes + SEM of 3 separate experiments performed in triplicate. * p < 0.05, relative to activity in NCI-N87 parental cells. **C**, NCI-N87 cells, control shRNA cells, Dvl2 shRNA cells, Dvl3 shRNA cells, and LRP6 shRNA cells were infected for 3 h with *H. pylori *P1 wt. Whole cell RIPA lysates were prepared. Western blot analysis was performed with antibodies as indicated. Diagram shows the relative amounts of Axin2. Densitometric values for Axin2 were normalized to GAPDH content in each sample and depicted relative to the values of the respective non-infected cells.

To study whether nuclear translocation of β-catenin in *H. pylori*-infected epithelial NCI-N87 cells depends on LRP6, Dvl2 or Dvl3, we performed an infection of stable knockdown cell lines deficient for LRP6, Dvl2 or Dvl3. β-catenin amounts were strongly reduced in cells deficient for LRP6, Dvl2, or Dvl3 compared to the control (Figure 4A). In addition, after different periods of time (3 h, 6 h, 24 h), infected cells were analysed for β-catenin transcriptional activity. Reporter construct activation was significantly reduced in cells either deficient for LRP6, Dvl2 or Dvl3 (Figure 4B). Further, we proved that reduced expression of Axin2 depends on LRP6, Dvl2, and Dvl3 regulation. In a Western blot analysis protein levels of Axin2 were clearly reduced in cells deficient either for LRP6, Dvl2 or Dvl3 (Figure 4C).

## Discussion

Germ line or somatic mutations in the Wnt/β-catenin signaling pathway cause several hereditary human diseases and are involved in the initiation or progression of a number of human cancers [18] mostly caused by inappropriate accumulation and activation of β-catenin. In addition, the development of human gastric cancer has been linked to altered expression or redistribution of β-catenin [2,19]. Recent studies could demonstrate that infection of epithelial cells with *H. pylori *stimulates transcriptional activity of β-catenin [20], but mechanistic insight for the underlying intracellular signaling leading to β-catenin induced transactivation of LEF/TCF dependent target gene expression remained unclear. First insight into the molecular machinery involved in *H. pylori *induced stabilization of β-catenin was given by Sokolova *et al*., demonstrating in primary epithelial Madin-Darby-Canine-Kidney (MDCK) cells T4SS-independent induction of the epidermal growth factor receptor (EGFR)-PI3K/Akt kinases after *H. pylori *infection, resulting in downstream GSK3β inhibition and reduced Ser/Thr phosphorylation of β-catenin [16]. In contrast to these data Tabassam *et al*. showed EGFR phosphorylation and Akt activation after *H. pylori *infection of gastric AGS and MKN tumor cell lines, which depends on the T4SS and the bacterial outer membrane protein OipA [21]. Other studies also using tumor cells reported that PI3K-mediated Akt phosphorylation depends on the bacterial protein VacA [22], or CagA [23].

In contrast to Serine/Threonine phosphorylations of the N-terminus of β-catenin, which are responsible for the degradation of the protein, regulation of the E-cadherin-catenin complex is regulated by phosphorylation of different tyrosine residues. Tyrosine phosphorylation of β-catenin at residues Tyr^489 ^and Tyr^654 ^disrupts binding to E-cadherin, and at Tyr^142 ^to α-catenin [6]. The receptor of hepatocyte growth factor, c-Met is able to phosphorylate β-catenin bound to E-cadherin at Tyr^142^, switching it from its adhesive to its transcriptional function [24]. Fyn is another tyrosine kinase which is able to phosphorylate β-catenin at the same residue [25]. Both proteins have been shown to become active after *H. pylori *infection [26,27].

In our work we present for the first time an analysis of the role of proximal Wnt signaling pathway components, namely LRP6, Dvl2, and Dvl3, in *H. pylori*-induced activation of β-catenin. Expression of Dvl1 was weak, therefore, we resigned to establish a knockdown cell line deficient for Dvl1. We used human NCI-N87 cells [28], which express full-length E-cadherin, and lack any known mutations in β-catenin, APC, or E-cadherin, and show no basal LEF/TCF activity, in contrast to AGS, MKN28, and MKN45 cell lines [29,30].

The Wnt signaling pathway co-receptor LRP6 is crucial for the stabilization of β-catenin [31] and its phosphorylation is one of the first key events in signal transduction after binding of Wnt ligands [32] or R-Spondin1 [33]. Recombinant Wnt3a was able to induce LRP6 phosphorylation in NCI-N87 cells, indicative of a functional Wnt signaling in these cells. We observed increased levels of phosphorylated LRP6 within 30 minutes post infection, which requires a functional T4SS, but not CagA or VacA. Infection of AGS cells confirmed these data as *H. pylori *induced a similar phosphorylation of LRP6 compared to NCI-N87 cells. Concerning the role of LRP6 in *H. pylori *infected cells we observed that the knockdown of LRP6 tremendously suppresses the amount of β-catenin, the LEF/TCF transactivation and the amounts of Axin 2 expression. Therefore, we suggest that LRP6 exerts an important role in the *H. pylori *dependent β-catenin regulation. A contributory role of LRP5 could be possible. Studying expression of mRNA we observed no differences between LRP6 and LRP5 in AGS and NCI-N87 cells (data not shown).

Overexpression of the cytoplasmic phosphoprotein Dvl has been shown to result in β-catenin signaling in *Drosophila *[34]. Moreover, phosphorylation of LRP6 is strictly dependent on Dvl [17,35] and Dvl proteins are thought to promote the co-recruitment of the Axin-GSK3β complex to the membrane, a step which seems to be required for maximal phosphorylation of LRP6. According to this, we found reduced levels of pLRP6 in NCI-N87 cells deficient for Dvl2 and Dvl3 after infection with *H. pylori*. Noteworthy, phosphorylation of LRP6 was not fully hindered with either Dvl2 or Dvl3 shRNA. The question remains open, if a double-knockdown of both proteins would completely reverse the observed LRP6 phosphorylation. Dvl proteins have been shown to show at least some redundancy [36], but not to a level one might expect considering the great homology between the members of the family. It rather seems to be the case that every Dvl protein has unique roles in cellular signaling [37].

Previous studies using different MKN gastric tumor cell lines could show nuclear β-catenin localization after infection with the NCTC11637 strain [20,38,39]. We used the NCI-N87 cell line as model and found elevated levels of β-catenin in nuclear fractions within 1 h post infection. In accordance, LEF/TCF transactivation was increased after infection with *H. pylori*, as previously demonstrated by others [16,20]. Upregulation of the well-established Wnt target gene Axin2 could be detected as soon as 3 h post infection. Axin2 can act as negative feedback molecule in the Wnt signaling pathway and is described to be regulated context-independently [9]. Interestingly, like in primary MDCK cells, an investigation using the MCF-7 breast cancer cell line showed a predominant cytoplasmic localization of β-catenin but absent LEF/TCF transactivation after prolonged infection. Additionally, the authors show a CagA-independent mechanism of *H. pylori *to deregulate cell adhesion through disconnection of the E-cadherin-catenin complex from the cytoskeleton [40]. Other reports suggest that overexpression of CagA is able to impair the complex formation of E-cadherin and β-catenin [20], or showed a CagA-independent effect on the E-cadherin-β-catenin complex [41] in *H. pylori*-infected cells. Beyond this, proteolytic cleavage of the extracellular domain of E-cadherin in *H. pylori*-infected epithelial cells contributes in a CagA-independent manner to the disintegration of adherence junctions [38,42].

Nuclear accumulation of β-catenin, LEF/TCF activation as well as target gene upregulation was clearly affected in cells deficient for LRP6, Dvl2, and Dvl3. Interestingly, a similarly efficient knockdown of Dvl2 when compared to knockdown of Dvl3 is less important for the activation of β-catenin in response to *H. pylori*. Future studies will have to determine if this difference is related to distinct functions of Dvl2 and Dvl3 in *H. pylori*-infected cells.

## Conclusion

**Figure 5:**
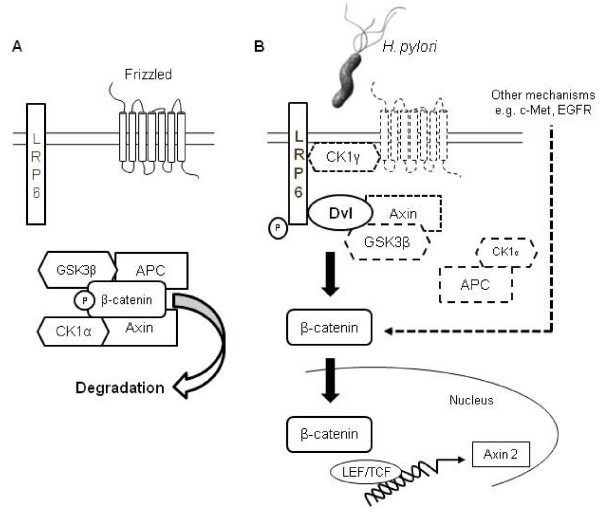
***H. pylori *induced activation of β-catenin involves LRP6 and Dvl**. **A **In non-infected cells, β-catenin is phosphorylated by a destruction complex consisting of Axin, APC, GSK3β and CK1α and subsequently degraded by the 26S proteasome. **B**, Infection of gastric epithelial cells with *H. pylori *induces LRP6 phosphorylation and activation of β-catenin in a LRP6- and Dvl-dependent manner, resulting in β-catenin translocation to the nucleus and induction of target gene expression (Axin2). Further, other factors, e.g. c-Met or EGFR contribute to β-catenin signaling in *H. pylori*-infected epithelial cells.

In our work we have investigated novel factors in β-catenin regulation in *H. pylori*-infected epithelial cells and show phosphorylation of LRP6 in a T4SS-dependent manner. Additionally, *H. pylori*-induced activation of β-catenin involves the Wnt signaling pathway components Dvl2, Dvl3 (Figure 5).

## Methods

### *H. pylori *strains

We used *H. pylori *wild type strain P1 and isogenic mutant strains cagA, virB7 and additionally P14 vacA strain. Bacteria were cultured for 48-72 h as described [26] and then added to epithelial cells at a multiplicity of infection of 100. Heat-killed *H. pylori *suspensions were prepared by incubating bacteria at 70°C for 10 min followed by 95°C for 5 min.

### Cell culture

NCI-N87 cells (ATCC, Manassas, USA) and AGS cells were maintained in RPMI 1640 (PAA, Cölbe, Germany) with 10% FCS and 1% penicillin/streptomycin in humid atmosphere at 37°C and 5% CO_2 _. Two days before infection/stimulation experiments 10^6 ^cells were seeded in 100 mm dishes in 8 ml of complete medium. 16 h prior to infection/stimulation medium was removed; cells were washed twice with PBS (w/o Ca^2+ ^and Mg^2+^) and fresh, serum- and antibiotic-free medium was added.

### Antibodies and chemicals

We used the following antibodies and chemicals: phospho-LRP6 (Ser1490), LRP6, Axin2, Dvl2, HDAC1, anti-mouse-HRP and anti-rabbit-HRP (Cell Signaling Technologies Inc.); Dvl3 and TfR1 (Santa Cruz Biotechnology); β-catenin (BD Transduction Laboratories); GAPDH (Chemicon International) Recombinant Wnt3a was purchased from R & D Systems.

Lentiviral constructs for the generation of stable knockdown cell lines for LRP6, Dvl2 and Dvl3 as well as a control construct were purchased from Open Biosystems.

### Transfection and luciferase reporter assay

NCI-N87 cells and the respective knockdown cell lines were seeded into 96-well plates at a density of 10^4 ^cells per well the day before transfection. 150 ng of total DNA were transfected per well with a ratio between firefly SuperTOPflash or SuperFOPflash (kindly provided by R. Moon, Seattle, USA) and renilla luciferase (Promega) plasmids of 9:1 using Lipofectamine LTX transfection reagent (Invitrogen). Luciferase activity was measured using the Dual-Luciferase Reporter Assay System (Promega) at Lumat LB 9507 luminometer (Berthold Technologies). All samples were assayed in triplicates.

### Preparation of cell lysates and subcellular fractions

Whole cell lysates were prepared using a modified RIPA buffer (50 mM Tris-HCl, pH 7.5, 100 mM NaCl, 5 mM EDTA, 1% Triton X-100, 10% glycerol, 10 mM K_2_HPO_4_, 0.5% Nonidet P40, 1 × protease inhibitor cocktail (Roche), 1 mM Na_3_VO_4_, 1 mM Na_2_MoO_4_, 20 mM NaF, 0,1 mM AEBSF, 20 mM β-glycerol-2-phosphate, 10 mM Na_4_P_2_O_7_). The homogenate was incubated 10 min on ice, drawn through a syringe (0.45 mm, 40.I.U., Omnifix, Braun, Germany). After another 10 min of incubation on ice, lysates were cleared by centrifugation for 10 min (12000 × g at 4°C). Aliquots of the respective samples were then boiled with sample buffer (50 mM Tris-HCl, pH 6.8, 2% SDS, 10% Glycerol, 100 mM DTT, 0.2% bromphenol blue) for 5 min at 95°C. Subcellular fractions were prepared using ProteoExtract Kit (Calbiochem) following the manufacturer's instructions.

### Establishment of stable knockdown cell lines

Lentiviral particles were produced as recently described [41]. In order to generate supernatants containing infectious lentiviral particles, HEK 293T cells were transfected with 3 μg pMD2.G, 5 μgpMD1g/pRRE and 2.5 μg pRSV-Rev of the lentiviral packaging vectors [41] together with the pGIPZ constructs for shRNAmir expression of Dvl2, Dvl3, LRP6, and a scrambled sequence from Open Biosystems. The supernatants were harvested 24 hrs post transfection, filtered (45 μm filter, Schleicher & Schuell, Dassel, Germany) and concentrated by centrifugation (19500 × g, 2 h at 12°C). The concentrated virus was added to NCI-N87 cells with 5 μg/ml Polybrene, and NCI-N87 cells were spin-infected. Stable cell lines were selected in 10 μg/ml Puromycin for one week.

### Western blot analysis

Protein samples were separated by SDS-PAGE and then transferred to Immobilon-P transfer polyvinylidene fluoride (PVDF) membrane (Millipore), blocked with 5% w/v milk or BSA/TBST followed by analysis with antibodies as indicated. Immunoreactivity was detected using the enhanced chemiluminiscence detection kit ECL™ (Amersham Pharmacia Biotech). Blots were semi-quantitatively evaluated by densitometric analysis. Protein bands on X-ray films were scanned using VersaDoc Imaging System (Bio-Rad), and analyzed using Quantity One software (Bio-Rad).

### Statistical analysis

Experiments were analysed using ANOVA and unpaired Student's t-test. The values of the experiments are expressed as means ± SEM. Statistical decisions were made with a critical probability of p = 5% without α-adjustment.

## Competing interests

The authors declare that they have no competing interests.

## Authors' contributions

TG performed most of the experiments, contributed to the design of the study and analysis of data, and drafted the manuscript. MF and ML generated the stable knockdown cell lines. UL and MN contributed to the design of the study, analysis of the data and editing of the manuscript. All authors read and approved the final manuscript.
